# The effectiveness of interventions for reducing subjective and objective social isolation among people with mental health problems: a systematic review

**DOI:** 10.1007/s00127-019-01800-z

**Published:** 2019-11-19

**Authors:** Ruimin Ma, Farhana Mann, Jingyi Wang, Brynmor Lloyd-Evans, James Terhune, Ahmed Al-Shihabi, Sonia Johnson

**Affiliations:** 1 Division of Psychiatry, University College London, 6th Floor, Maple House, 149 Tottenham Court Road, London, W1T 7NF England, UK; 2 UCL Medical School, University College London, 74 Huntley Street, London, WC1E 6BT England, UK; 3 Camden and Islington NHS Foundation Trust, St. Pancras Hospital, 4 St. Pancras Way, London, NW1 0PE England, UK

**Keywords:** Loneliness, Perceived social support, Objective social isolation, Mental health, Systematic review, Intervention

## Abstract

**Purpose:**

Subjective and objective social isolation are important factors contributing to both physical and mental health problems, including premature mortality and depression. This systematic review evaluated the current evidence for the effectiveness of interventions to improve subjective and/or objective social isolation for people with mental health problems. Primary outcomes of interest included loneliness, perceived social support, and objective social isolation.

**Methods:**

Three databases were searched for relevant randomised controlled trials (RCTs). Studies were included if they evaluated interventions for people with mental health problems and had objective and/or subjective social isolation (including loneliness) as their primary outcome, or as one of a number of outcomes with none identified as primary.

**Results:**

In total, 30 RCTs met the review’s inclusion criteria: 15 included subjective social isolation as an outcome and 11 included objective social isolation. The remaining four evaluated both outcomes. There was considerable variability between trials in types of intervention and participants’ characteristics. Significant results were reported in a minority of trials, but methodological limitations, such as small sample size, restricted conclusions from many studies.

**Conclusion:**

The evidence is not yet strong enough to make specific recommendations for practice. Preliminary evidence suggests that promising interventions may include cognitive modification for subjective social isolation, and interventions with mixed strategies and supported socialisation for objective social isolation. We highlight the need for more thorough, theory-driven intervention development and for well-designed and adequately powered RCTs.

## Introduction

Subjective social isolation and objective social isolation are conceptually distinct [1] and often only moderately correlated [2]. The terms loneliness and perceived social support both refer to people’s subjective perception of their social world (i.e. subjective social isolation) [1, 3]. Loneliness is defined as the unpleasant experience that occurs when there is a subjective discrepancy between desired and perceived availability and quality of social interactions [4]. Perceived social support is the self-rated adequacy of the social resources available to a person [5]. Well-established and widely used measures of loneliness are available, such as the UCLA Loneliness Scale [6]. Objective social isolation, meanwhile, involves having little social contact with other people [7] and can be objectively defined based on quantitative measures of social network size or the frequency of social contacts with others [8]. A summary table of commonly used measures of subjective and objective social isolation is provided in Appendix 1.

In a UK survey, approximately one in five of the general population reported being lonely in the preceding 2 weeks [9]. For people with mental health problems, the odds of being lonely were eight times greater than for the general population, and the odds were increased 20-fold for those with two or three diagnoses (e.g. depression and schizophrenia), compared to those without any diagnosis [10]. Objective social isolation, including having fewer friends [11] and being less likely to date [12], is also more common among people with mental health problems than in the general population. Loneliness has adverse health effects, such as an impaired immune system [13], elevated blood pressure [14], depression [15], and cognitive decline [16]. Moreover, loneliness is associated with poorer quality of life [17] and personal recovery [18], and with more severe mental health symptoms [19]. Similarly, a number of negative health outcomes have been found to be associated with objective social isolation, for example, increased all-cause mortality rate [20], poor physical health outcomes [21, 22], worse psychotic symptoms [23, 24], depressive symptoms [24], and higher risk of dementia [25]. Conversely, social support that is perceived as sufficient is associated with less severe psychiatric symptoms, higher functioning, better personal recovery, greater self-esteem and empowerment, and improved quality of life [26]. These associations between subjective and objective social isolation and poorer outcomes [27–30] make interventions designed to alleviate social isolation of high interest. Subjective social isolation has recently been increasingly recognised as a treatment priority for people with serious mental illness [31]. By targeting both subjective and objective social isolation as main outcomes in the current review, we aimed to establish the extent of the current evidence base for interventions for each of these potential treatment targets and to understand the similarities or differences between the characteristics of interventions that work for subjective and for objective social isolation.

Some authors have previously systematically reviewed interventions for subjective social isolation [30, 32–35] and objective social isolation [36–38] (Appendix 2). The most recent systematic review focused on subjective social isolation among people with mental health problems was published in 2005 [35]. Three more recent systematic reviews focused on aspects of objective social isolation: one reviewed interventions to increase network size in psychosis [37] and the other two examined interventions targeting social participation in people with mental health problems [36, 38]. Thus, there is no up-to-date systematic review of evidence for a full range of interventions to alleviate subjective and/or objective social isolation among people with a mental health diagnosis.

Masi’s meta-analysis in 2011 [34] has been considered one of the most comprehensive reviews to date examining interventions for loneliness, identifying four main types of intervention. However, Masi’s review included only 20 RCTs and included all populations, not only people with mental health problems. Thus, our paper adds to knowledge from Masi’s review by providing an up-to-date synthesis of interventions for loneliness in people with mental health problems, using a typology of interventions targeting loneliness and related constructs recently developed by Mann and her team [39]. This typology distinguishes among the following: (1) interventions involving changing maladaptive cognitions about others (e.g. cognitive-behavioural therapy or reframing); (2) social skills training and psychoeducation programmes (e.g. family psychoeducation therapy); (3) supported socialisation (e.g. peer support groups, social recreation groups); and (4) wider community approaches (e.g. social prescribing and asset-based community development approaches). These community approaches maximise individuals’ engagement with social resources and/or aim to develop social resources at the level of whole communities.

## Methods

We conducted the current systematic review to evaluate the evidence for the effectiveness of interventions designed to alleviate subjective social isolation (including loneliness and perceived social support) and/or objective social isolation among people with mental health problems.

### Inclusion criteria

#### Types of study

Only randomised controlled trials (RCTs) were included, with no restrictions on publication dates, the country of origin or language.

#### Participants

People primarily diagnosed with mental health conditions were included, including depression, anxiety, post-traumatic disorder, psychosis/schizophrenia or bipolar disorders. Any method of identifying or diagnosing people as mentally ill was acceptable. There was no restriction on the age of the participants. However, studies where the samples were people with a primary diagnosis of intellectual disability, autistic spectrum disorders, dementia, any other organic illnesses, substance misuse or physical health problems were excluded, even if some had comorbid mental health diagnoses.

#### Interventions

This review included interventions which were designed to alleviate subjective or/and objective social isolation for people with mental health problems. Papers were included if subjective or objective social isolation was a primary outcome and excluded if they were secondary outcomes, with another clearly specified primary outcome. Trials were also included if a clear distinction was not made between primary and secondary outcomes, with subjective and/or objective social isolation as one of a number of main outcomes.

#### Comparison

We included trials where the control group received treatment-as-usual, however defined, no treatment or a waiting-list control. We also included trials which compared different active treatment groups.

#### Outcomes

The primary outcomes were subjective social isolation (including loneliness and perceived social support) and objective social isolation. End-of-treatment outcomes, medium-term outcomes (i.e. up to one year beyond the end-of-treatment time point) and longer-term follow-up outcomes (i.e. more than one year beyond the end-of-treatment time point) were reported separately. The following secondary outcomes were also examined: health status, quality of life, and service use.

### Search strategy

Three databases were systematically searched for relevant literature: Medline, Web of Science and PsycINFO. Three groups of search terms were combined: (1) subjective and objective social isolation (e.g. loneliness); (2) mental disorders (e.g. psychosis, depression, post-traumatic stress disorder) and (3) trials (e.g. RCT). For detailed search terms, please see Appendix 3. Reference lists from included studies, relevant systematic reviews, and meta-analyses were hand-searched. Grey literature was searched through OpenGrey by using keywords ‘loneliness’, ‘perceived social support’ and ‘social isolation’.

### Data extraction

RM and FM reviewed all titles and abstracts, AA screened half of the papers we retrieved, and final decisions regarding whether a paper should be included or not were made by all three independent reviewers. The primary reviewer (RM) reviewed all full-text papers retrieved, and inter-rater reliability was also evaluated as good between reviewers during the screening process. The final list of included papers was confirmed only when RM, FM, and AA agreed on all papers. Any differences were resolved in consultation with a further independent reviewer (SJ). Data were extracted by RM and FM by using a standardised form developed for the review, including items related to publication year and country, study design, experimental settings, participants, the nature of the intervention, follow-up details, primary and secondary outcomes, any exclusions of participants, and the reasons for these, confounders, and risk of bias.

### Quality assessment

The Cochrane Risk of Bias tool [40] was used for the quality assessment. Each included paper was assessed by two reviewers (RM and FM/JT) regarding the following domains: sequence generation, allocation concealment, blinding of participants, personnel and outcome assessors, incomplete outcome data, selective outcome reporting and other sources of bias. For each paper, a final decision for each domain was made only if both assessors agreed. If there was disagreement, a third independent assessor (SJ) was consulted.

### Synthesis plan

A narrative synthesis was conducted for this systematic review based on the principles from the ESRC’s Guidance on the Conduct of Narrative Synthesis in Systematic Reviews [41]. The included trials were grouped into three categories: (1) trials that included subjective social isolation as an outcome (primary or one of several, with none specified as primary); (2) trials that included objective social isolation as an outcome; and (3) trials that included both outcomes. Due to the expected heterogeneity in samples and intervention types from this broad review, meta-analysis was judged to be inappropriate.

## Results

**Fig. 1:**
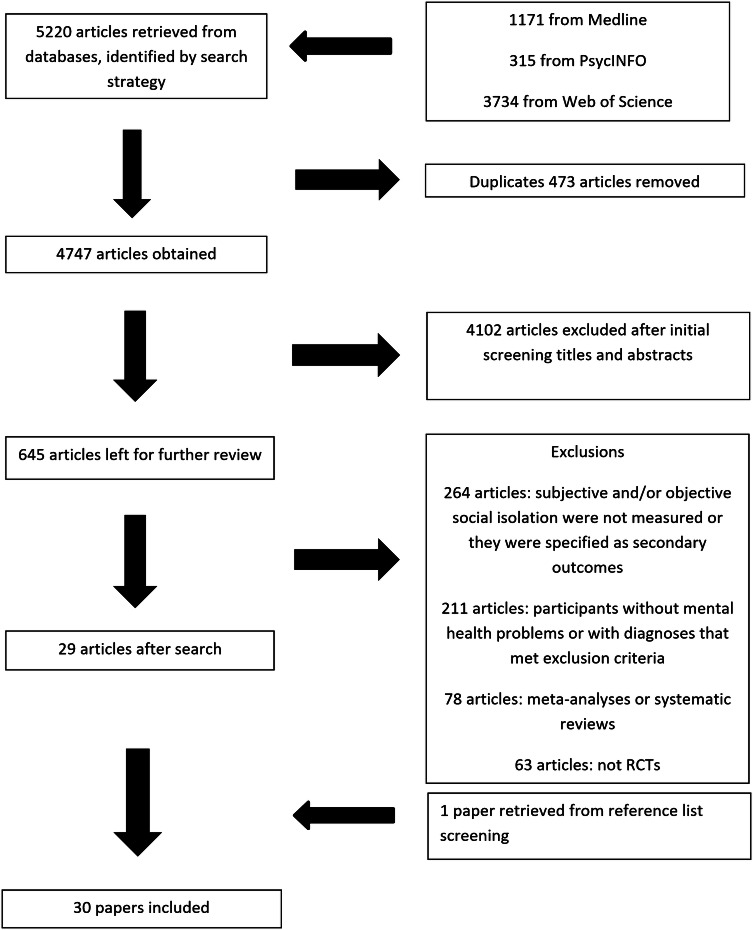
PRISMA diagram for literature search

The initial literature search retrieved 5220 papers in total, of which 30 were found to be eligible for inclusion. The PRISMA flow diagram (Fig. 1) shows details of the screening process.

The 30 trials involved 3080 participants in total, with individual trial sample sizes ranging from 21 to 357. Nineteen trials had fewer than 100 participants. The median number was 88, and the interquartile range (IQR) was 104. Authors from nine trials specified sample size calculations. The search was conducted in July 2017 and all trials were published between 1976 and 2016. Thirteen trials were conducted in the US, 11 in Europe, 3 in Israel, 2 in China, and 1 in Canada. Thirteen interventions were conducted individually, nine interventions were delivered in groups, four involved individual and group support, and four were implemented online. Ten trials compared different active treatments, four of which had no control group. The remaining 20 trials compared intervention groups with a control group: 13 involved treatment-as-usual groups, 5 involved waiting-list controls, and 2 involved no-treatment controls.

### Interventions to reduce subjective social isolation

Fifteen trials included subjective social isolation as primary outcome, or as one of several outcomes with none specified as primary (Table 1).

**Table 1:** Trials that included subjective social isolation as outcome

Main author, sample and setting	Intervention categorisation	Intervention name and duration	Follow-up	Social isolation and other outcome measures	Subjective social isolation outcomes
Group-based intervention					
Hasson-Ohayon [42]—210 adults with severe mental illness Psychiatric community rehabilitation centre in Israel (secondary care setting)	Psychoeducation, social skills training	Illness Management and Recovery Programme vs. treatment-as-usual control group Duration: 8 months	End-of-treatment follow-up (8 months)	Subjective social isolation outcome: the Multidimensional Scale of Perceived Social Support (MSPSS) [57] Other outcome: personal recovery	No significant changes in perceived social support for either group. *p* > 0.05^a^
Silverman [43]—96 adults with varied Axis I diagnoses Acute care psychiatric unit in a University hospital, the Midwestern region in the US (secondary care setting)	Psychoeducation	Live educational music therapy (condition A), recorded educational music therapy (condition B), education without music (condition C), recreational music therapy without education (condition D) Duration: 24 weeks	End-of-treatment follow-up (24 weeks)	Subjective social isolation outcome: the MSPSS [57]	No significant between-group difference in total perceived social support for condition A vs. B, condition A and B vs. condition C, as well as for condition A and B vs. D (all *p* > 0.05) (*F* (3.87) = 1.50, *p* = 0.22) Partial effect size = 0.028 for support from significant other, 0.015 for support from family, 0.094 for support from friends, and 0.049 for total support Only a significant between-group difference between condition A vs. D on a friend subscale, 95% CI (0.47, 10.40), adjusted *p* = 0.02, mean difference = 5.34
Boevink [44] - 163 adults with mental illness Mental health care organisations (community treatment team and sheltered housing organisations) in the Netherlands (secondary care setting)	Supported socialisation	Toward Recovery, Empowerment and Experiential Expertise (TREE) + care-as-usual vs. care-as-usual control group Duration: 104 weeks for early starters and 52 weeks for late starters	1 medium-term follow-up: 12 months (post-baseline) 1 long-term follow-up: 24 months (post-baseline)	Subjective social isolation outcome: the De Jong-Gierveld Loneliness Scale [58] Other outcomes: quality of Life; psychiatric symptoms	No between-group difference in loneliness, 95% CI (− 0.31, 0.30) (effect size linear tread *B* = − 0.053, *p* = 0.98), standardised effect size was − 0.001 for each year of exposure to TREE programme
Eggert [45]—105 high school students with poor grades (moderate or severe depression) 5 urban high schools in the US (general population setting)	Supported socialisation, social skills training and wider community approaches	Assessment protocol plus 1-semester Personal Growth Class (PGCI) vs. Assessment protocol plus a 2-semester Personal Growth Class (PGCII) vs. an assessment protocol-only Duration: 5 months or 90 class days in length for PGCI, and 10 months or 190 class days in length for PGCII	2 medium-term follow-ups: 5 and 10 months (post-baseline)	Subjective social isolation outcomes: perceived social support was measured by calculating average ratings across 6 network support sources. Instrumental and expressive support provided by each network support source (e.g. family, friends) was also rated on a scale Other outcome: depressive symptoms	All 3 groups showed increased network social support *F* linear (1,100) = 32.08, *p* < 0.001 No significant between-group difference between all groups *F* linear (1,100) = 1.98, *p* = 0.143
Individual-based intervention					
Zang [46]—30 adults aged 28–80 with post-traumatic stress disorder (PTSD) Beichuan County in China (general population setting)	Changing cognitions	Narrative Exposure Therapy (NET) vs. Narrative Exposure Therapy Revised (NET-R) vs. waiting-list control group Duration: 2 weeks for NET and 1 week for NET-R group	End-of-treatment follow-up (2 weeks for NET, 1 week for NET-R) 2 medium-term follow-ups: 1 week (for NET) or 2 weeks (for NET-R), and 3 months	Subjective social isolation outcome: the MSPSS [57] Other outcomes: anxiety and depressive symptoms; PTSD symptoms	Both NET and NET-R showed effects on perceived social support after treatment, but no significant between-group difference between the two groups (*F *(2,26) = 0.14, *p* > 0.05) No significant between-group difference between either treatment group (NET and NET-R) and the waiting-list control in perceived social support (both *p* > 0.05)
Zang [47]—22 adults aged 37–75 with PTSD Beichuan Country in China (general population setting)	Changing cognitions	NET intervention vs. waiting-list control group Duration: 2 weeks	End-of-treatment follow-up (2 weeks) 2 medium-term follow-ups: 2 weeks, and 2 months	Subjective social isolation outcome: the MSPSS [57] Other outcomes: subjective level of distress; depressive symptoms	No significant between-group difference in perceived social support (*F *(1,19) = 4.25, *p* = 0.05, *d* = 0.33)
Gawrysiak [48]—30 adults aged ≥ 18 with depression A public Southeastern University in the US (general population setting)	Psychoeducation, social skills training and supported socialisation	Behavioural Activation Treatment for Depression (BATD) vs. no-treatment control group Duration: single session lasted 90 min	1 medium-term follow-up: 2 weeks	Subjective social isolation outcome: the MSPSS [57] Other outcomes: depressive symptoms; anxiety symptoms	No significant between-group difference in perceived social support (*F *(1,28) = 3.11, *p* = 0.08, *d* = 0.70)
Conoley [49]—57 female psychology undergraduate students with moderate depression University Psychology department in the US (general population setting)	Changing cognitions	Reframing vs. self-control vs. waiting-list control group Duration: 2 weeks	End-of-treatment follow-up (2 weeks) 1 medium-term follow-up: 2 weeks	Subjective social isolation outcome: the Revised University of California Los Angeles (UCLA) Loneliness Scale [59]; The Causal Dimension Scale [60] Other outcome: depressive symptoms	No significant treatment effect was found (*F *(2,108) = 0.60, *p* > 0.05^b^)
Bjorkman [50]—77 adults aged 19–51 with severe mental illness Case management service in Sweden (secondary care setting)	Social skills training	The case management service vs. standard care Duration: unclear	2 long-term follow-ups: 18 and 36 months	Subjective social isolation outcome: the abbreviated version of the Interview Schedule for Social Interaction (ISSI) [61] Other outcomes: psychiatric symptoms; quality of life; use of psychiatric services	No significant between-group difference between two groups in social outcomes (*p* > 0.05)^c^
Mixed-format (group- and individual-based)					
Mendelson [51]—78 depressed women aged 14–41 who were either pregnant or had a child less than 6 months old Home visiting programme in Baltimore City in the US (general population setting)	Changing cognitions	Standard home visiting services + The Mother and Babies (MB) course vs. standard home visiting services + information on perinatal depression Duration: 6 weeks	End-of-treatment follow-up (6 weeks) 2 medium-term follow-ups: 3 and 6 months	Subjective social isolation outcome: the Interpersonal Support Evaluation List (ISEL) [62]	No significant between-group difference in perceived social support, *β* = 6.67, SE = 0.03, *p* < 0.10^d^
Masia-Warner [52]—35 high school students with social anxiety disorder 2 parochial high schools in New York, US (general population setting)	Psychoeducation/social skills training, supported socialisation and changing cognitions	Skills for Social and Academic Success vs. waiting-list control group Duration: 3 months	End-of-treatment follow-up (3 months) 1 medium-term follow-up: 9 months	Subjective social isolation outcome: Loneliness Scale [63] Other outcomes: anxiety symptoms; social phobic symptoms; depressive symptoms	No significant treatment effect, effect size = 0.20^e^, *p* > 0.05
Online intervention					
Kaplan [53]—300 adults with schizophrenia spectrum or affective disorder Online in the US (general population setting)	Supported socialisation	Experimental peer support listserv vs. experimental peer support bulletin board vs. waiting-list control group Duration: 12 months	2 medium-term follow-ups: 4 and 12 months (post-baseline)	Subjective social isolation outcome: the Medical Outcomes Study (MOS) Social Support Survey [64] Other outcomes: personal recovery; quality of life; psychiatric symptoms	No significant between-group difference on MOS (*F *(1,298) = 0.08, *p* = 0.93), also not significant when two experimental groups compared to the control group separately (*p* > 0.05)
Rotondi [54]—30 patients aged ≥ 14 with schizophrenia or schizoaffective disorder In- and out-patient psychiatric care units and psychiatric rehabilitation centres in Pittsburgh, Pennsylvania (secondary care setting)	Psychoeducation	Telehealth intervention vs. usual care group Duration: unclear	2 medium-term follow-ups: 3 and 6 months (post-baseline)	Subjective social isolation outcome: the informational support and emotional support subscales of the instrument that was developed by Krause and Markides [65]	No significant between-group difference on perceived social support (*F *(1,27) = 3.79, *p* = 0.062)
O’Mahen [55]—83 women aged > 18 with major depressive disorder (MDD) Online in the UK (general population setting)	Psychoeducation and supported socialisation	Netmums Helping with Depression (HWD) vs. treatment-as-usual control group Duration: unclear	End-of-treatment follow-up (unclear) 1 medium-term follow-up: 6 months	Subjective social isolation outcome: the Social Provision Scale [66] Other outcomes: depressive symptoms; anxiety symptoms	No significant between-group difference in perceived support between the intervention and control group (95% CI 1.02, − 0.02), medium effect size = 0.50 (*p* = 0.27)
Interian [56]—103 veterans with PTSD Online in the US (primary care setting)	Psychoeducation and changing cognitions	The Family of Heroes intervention vs. no-treatment control group Duration: unclear	1 medium-term follow-up: 2 months (post-baseline)	Subjective social isolation outcome: the family subscale of the MSPSS [57]	Intervention group reported a higher chance of having a decreased perceived family support over time than the control group (*p* = 0.04)^f^

^a^Effect size, confidence interval and actual *p* value not available in the paper

^b^Confidence interval and actual *p* value not available in the paper

^c^Effect size, confidence interval and actual *p* value not available in the paper

^d^Effect size and confidence interval not available in the paper

^e^Confidence interval and actual *p* value not available in the paper

^f^Effect size not available in the paper

Two trials included only end-of-treatment outcomes [42, 43]. The follow-up period of the other 13 trials ranged from 1 week to 36 months beyond the end of treatment. The Multidimensional Scale of Perceived Social Support (MSPSS) and UCLA Loneliness Scale were frequently administered. The measures used in 14 trials have been shown to have good validity and reliability, but one trial [44] did not use a well-established scale. Nine trials involved people with common mental illnesses (e.g. depression), three involved people with severe mental illnesses (e.g. schizophrenia), and three included people with a variety of mental health diagnoses. The majority of the trials had small sample sizes (< 100); only four trials had more than 200 participants. Five trials included a sample size calculation.

Three trials involved online interventions, one trial combined online intervention and telephone support, four trials implemented face-to-face group intervention, five used face-to-face individual therapy, and the remaining two combined both group and individual formats. Interventions in two trials involved supported socialisation, four trials evaluated psychoeducation/social skills training, four had cognition modification elements, and five trials mixed different intervention types. The duration of the interventions ranged from 1 week to 104 weeks, while such information was missing in four trials and one trial involved only a single intervention session (Appendices 4 and 5).

Regarding quality assessment, method of randomisation was mentioned in half of the trials. Information on allocation concealment, missing data, and blinding were not sufficiently described in most trials. For detailed quality assessments, please see Appendix 6.

Of the ten trials that compared an active intervention with a control group [42, 44, 47, 48, 50–52, 54–56], none of the authors reported a significant between-group difference. In the five trials comparing at least two different active interventions [43, 45, 46, 49, 53], only Silverman and colleagues [43] found significant between-group differences, reporting a greater improvement in perceived social support from friends (measured by MSPSS friend subscale) in an intervention group involving both music therapy and psychoeducation than in other treatment groups (e.g. music alone). However, differences were not found in other outcomes and this trial did not involve a waiting-list or treatment-as-usual control. As most trials had small samples and lacked sample size calculations, clear conclusions cannot be drawn from negative results.

Eleven out of 15 trials included measures of other relevant outcomes [42, 44–50, 52, 53, 55]. Of these 11 trials, positive outcomes were reported by authors of seven trials. Improved depressive symptoms were reported in trials of interventions with mixed strategies with the following participant groups: adults in the community [48], urban high schoolers [45], and women with major depressive disorders [55]. Another mixed intervention had an effect on social avoidance and social phobia among high school students [52]. A diagnostically mixed participant group exhibited improved progress towards personal recovery and personal goals with psychoeducation/social skills training [42], and a mixed sample who received supported socialisation [44] also reported an improvement in quality of life. However, results on some outcomes in some of the trials did not show significant differences: an intervention with positive results for depression did not improve anxiety [48]; a case management service was not associated with any change in quality of life [50]; an online intervention for people with schizophrenia did not lead to any differences in quality of life or symptoms [53].

### Interventions to reduce objective social isolation

Eleven trials included objective social isolation as primary outcome, or as one of several outcomes with none identified as primary (Table 2).

**Table 2:** Trials that included objective social isolation as outcome

Main author, sample and setting	Intervention categorisation	Intervention name and duration	Follow-up	Objective social isolation and other outcome measures	Objective social isolation outcome
Group-based intervention					
Atkinson [67]—146 registered patients with schizophrenia Community clinic in south Glasgow, UK (secondary care setting)	Psychoeducation	The education group vs. waiting-list control group Duration: 20 weeks	End-of-treatment follow-up (20 weeks) 1 medium-term follow-up: 3 months	Objective social isolation outcome: a modified Social Network Schedule (SNS) [78] Other outcomes: quality of life; psychiatric symptoms; overall functioning	Significant between-group difference in the total number of contacts after the intervention (*t* = 4.4, *p* < 0.001) and at follow-up (*t* = 3.6, *p* < 0.001). Significant between-group difference in the number of confidants after the intervention (*t* = 3, *p* = 0.004) and at follow-up (*t* = 2.8, *p* = 0.006) Significant between-group difference over time from post-group (*t* = 2.8, *p* = 0.007) to follow-up (*t* = 2.5, *p* = 0.02)
Hasson-Ohayon [68]—55 adults aged 21–62 with various mental illness 3 psychiatric rehabilitation agencies and the University Community Clinic in Bar-Ilan University, Israel (secondary care setting)	Wider community approaches, psychoeducation/social skills training and changing cognitions	Social Cognition and Interaction Training (SCIT) + social mentoring vs. social mentoring only Duration: unclear	1 medium-term follow-up: 6 months	Objective social isolation outcome: the socio-engagement and interpersonal-communication subscales of the Social Functioning Scale (SFS) [79]	Experimental group showed significantly more improvement in social engagement than the controls (*F* (1,53) = 28.9, *p* < 0.001, effect size = 0.35), but no significant between-group difference for the interpersonal communication subscale (*F* (1,53) = 0.55, *p* = 0.464, effect size = 0.01)
Bøen [69]—138 seniors with mild depression 2 Municipal districts in eastern and western Oslo, Norway (general population setting)	Supported socialisation and wider community approaches	A preventive senior centre group programme vs. waiting-list control Duration: 1 year	End-of-treatment follow-up (1 year)	Objective social isolation outcome: the Oslo-3 Social Support Scale [80]^a^ Other outcomes: depressive symptoms; life satisfaction	Both groups had an increased level of social support, but greater improvement in the intervention group than the control group, *d* = 0.12, 95% CI (− 0.47, 0.81).
Individual-based intervention					
Solomon [70]—96 adults with schizophrenia or major affective disorders A community mental health centre in the US (secondary care setting)	Supported socialisation and wider community approaches	Consumer management team vs. non-consumer management team Duration: unclear	2 medium-term follow-ups: 1 month and 1 year (post-baseline)	Objective social isolation outcomes: family and social contacts; Pattison’s Social Network Scale [81] Other outcomes: use of services; quality of life; psychiatric symptoms	No significant between-group difference in social networks (*p* > 0.05)^b^ On average, participants identified 2.72 persons in their social network, 1.55 positive network members and 1.60 family members
Aberg-Wistedt [71]—40 adults with schizophrenia or long-term psychotic disorder The Kungsholmen sector in Stockholm, Sweden (secondary care setting)	Psychoeducation/social skills training	The intensive case management programme vs. standard services Duration: 2 years	One long-term follow-up: 2 years (post-baseline)	Objective social isolation outcome: the number of people in participants’ social life was measured by a standardised procedure developed from work with child psychiatric patients [82] Other outcomes: quality of life; service use	Social network of the experimental group increased, while it decreased for the control group, but no significant between-group difference (*p* > 0.004)^c^
Stravynski [72]—22 adults aged 22–57 with diffuse social phobia and avoidant personality disorder The Maudsley hospital in London, UK (secondary care setting)	Social skills training and changing cognitions	Social skills training vs. Social skill training + cognitive modification Duration: 14 weeks	End-of-treatment follow-up (14 weeks) 1 medium-term follow-up: 6 months	Objective social isolation outcome: objective social isolation subscale of the Structured and Scaled Interview to Assess Maladjustment (SSIAM) [83] Other outcome: depressive symptoms	No significant between-group difference in social isolation, all groups reported less experience of social isolation over time *p* > 0.05^d^
Terzian [73]—357 adults aged < 45 diagnosed as on the schizophrenia spectrum by the ICD-10th 47 community mental health services (SPT) in Italy (secondary care setting)	Supported socialisation and wider community approaches	Social network intervention + usual treatments vs. usual treatments Duration: 3–6 months	1 medium-term follow-up: 1 year (post-baseline) 1 long-term follow-up: 2 years (post-baseline)	Objective social isolation outcome: social networks measured by different parameters of relationships were assessed, all were summarised into a score Other outcomes: psychiatric symptoms; hospitalisation over the follow-up year	In this paper, a social network improvement was defined as an increase in the number, frequency, importance or closeness of relationships, and an overall social network improvement was definied as an improvement in intimate or working relationships. Significant between-group differences in the improvement of social network and overall social network improvement were found An improvement in social network was found at year 1 in 25% of patients in control group and 39.9% of patients in the experimental group (OR 2.0, 95% CI 1.3–3.1; AOR 2.4, 95% CI 1.4–3.9) At year 1, an overall social network improvement was reported for 30.8% of the routine group and 44.5% of the experimental group (OR 1.8, 95% CI 1.2–2.8; AOR 2.1, 95% 1.3–3.4) These differences remained significant at year 2 for social network improvement (31.5% in the control group and 45.5% in the experimental group, OR 1.8, 95% CI 1.1–2.8; AOR 2.1, 95% CI 1.3–3.5) and for overall social network improvement (33.3% for routine group, 47.9% for the experimental group, OR 1.8, 95% CI 1.2–2.9; AOR 2.2, 95% CI 1.3–3.5)
Solomon [74]—96 adults with schizophrenia or major affective disorders A community mental health centre in the US (secondary care setting)	Supported socialisation and wider community approaches	Consumer case management team vs. nonconsumer management team Duration: 2 years	2 medium-term follow-ups: 1 month and 1 year (post-baseline) 1 long-term follow-up: 2 years (post-baseline)	Objective social isolation outcome: Pattison’s Social Network [81] Other outcomes: quality of life; psychiatric symptoms	No significant between-group difference in social outcome; also no significant time and condition effect (*F *(12,78) = 1.19, *p* > 0.05^e^)
Marzillier [75]—21 adults aged 17–43 with a diagnosis of personality disorder or neurosis The Maudsley Hospital in London, UK (secondary care setting)	Social skills training and changing cognitions	Systematic Desensitisation (SD) vs. Social Skills Training (SST) vs. waiting-list control Duration: 3.5 months	End-of-treatment follow-up (3.5 months) 1 medium-term follow-up: 6 months	Objective social isolation outcome: Revised-Social Diary and Standardised interview Schedule [75] Other outcomes: anxiety disorders; mental state; personality assessment	No between-group difference between SST and SD in social activities and social contacts (*p* > 0.05) SST had a greater improvement in the range of social activities (*F* (1,18) = 7.56, *p* < 0.025) and social contacts (*F* (1,18) = 9.47, *p* < 0.0.01) than the waiting-list group SD had a greater increase in social contacts than the waiting-list group (*F *(1,18) = 12.46, *p* < 0.001)
Cole [76]—32 adults with major depression, dysthymic disorder or other affective disorder St. Mary’s Hospital in Montreal, Canada (primary care setting)	Nonspecific type (intervention group received a psychaitric assessment at home, compared to a standard treatment group who received an assessment at clinic)	Home assessment group vs. clinic assessment group (treatment-as-usual) Duration: unclear	3 medium-term follow-ups: 4, 8 and 12 weeks (post-baseline)	Objective social isolation outcome: Social Resources (SR) subscale from The Older Americans Research and Service Centre Instrument (OARS) [84] Other outcomes: mental state; psychiatric symptoms	No significant between-group differences in social resources (*p* > 0.05)^f^
Mixed format (group- and individual-based)					
Rivera [77]—203 adults with a psychotic or mood disorder on axis I An inpatient unit in a city hospital in New York, US (secondary care setting)	Supported socialisation	Peer-assisted care vs. Nonconsumer assisted vs. standard care vs. clinic-based care Duration: unclear	2 medium-term follow-ups: 6 and 12 months (post-baseline)	Objective social isolation outcome: a modification of the Pattison Network Inventory [85] Other outcomes: quality of life; psychiatric symptoms	Only peer-assisted group showed an increase in social contacts from baseline to 12-month follow-up (*F *(2, 118) = 7.25, *p* < 0.01, effect size = 0.11) No significant between-group difference in other network measures (*p* > 0.05)

^a^Due to the fact that the Oslo-3 scale focuses primarily on the practical aspects of social support, Bøen’s study was considered as a study only of objective social isolation

^b^Effect size, confidence intervals, and actual *p* value not available in the paper

^c^Effect size, confidence intervals, and actual *p* value not available in the paper; the significant level used in this study was *p* < 0.004

^d^Effect size not available in the paper

^e^Effect size, confidence interval, and actual *p* value not available in the paper

^f^Effect size, confidence interval, and actual *p* value not available in the paper

Of 11 trials, one trial [67] only included end-of-treatment outcomes. The follow-up period of the other ten trials ranged from 4 weeks to 2 years beyond the end-of-treatment. In eight trials, validated objective social isolation scales were used. In one trial, objective social isolation was measured by summarising the number, frequency, and type of social connections [73], one trial combined both methods [70], and we could not establish the validity of the measure used in another trial because too little detail was provided [71]. Three trials included people with common mental health problems, six trials involved people with severe mental illnesses, and two trials included diagnostically mixed populations. Most trials involved fewer than 100 participants, and only two had more than 200. Three trials included a sample size calculation.

Seven trials were implemented in an individual format, three were group-based interventions, and one involved both group and individual sessions, plus telephone support. Two trials involved a psychoeducation component/social skills training, one included supported socialisation opportunities, the intervention type of another trial was unclear, and the other seven trials involved interventions with multiple components. The duration of the interventions ranged from 12 weeks to 2 years where this was specified, but such information was not given in four trials (Appendices 4 and 5).

A description of the randomisation process was only included in three trials. Allocation concealment detail was described in five trials. Authors of seven trials did not report how they dealt with missing data. For detailed quality assessments, please see Appendix 6.

Of the six trials that compared an active treatment group with a control group [67–69, 71, 73, 76], findings of four trials suggested superior outcomes for their intervention groups over their control groups on objective social isolation measures: a psychoeducation programme for adults with schizophrenia [67], a social network intervention for people diagnosed with schizophrenia spectrum disorders [73], a preventive senior centre group for seniors with mild depression [69], and Social Cognition and Interaction Training (SCIT) for patients with various diagnoses [68]. One trial involving social education for people with schizophrenia and one trial involving home assessment teams for people with mood disorders did not lead to any improvements in objective social isolation [71, 76].

Of the five trials that compared different active interventions [70, 72, 74, 75, 77], positive findings were reported in two trials. One trial included systematic desensitisation and social skills training interventions: both were found to be superior to the control group for increasing social contacts in a sample with personality or mood disorders, although there was no between-group difference between the two active treatment groups [75]. Rivera and colleagues [77] reported an increased contact with staff for participants receiving a consumer-provided programme, compared to non-consumer support. However, Solomon and colleagues [70, 74] also compared consumer versus non-consumer provided mental health care in their two studies and found no significant differences between the groups, or compared to a control group, in social network size or clinical outcomes. Stravynski and colleagues examined whether adding a cognitive modification component to social skills training for people with social phobia and/or avoidant personality disorders improved its effectiveness [72], but found no significant difference between groups. Therefore, the overall evidence regarding the effectiveness of consumer-provided intensive case management for objective social isolation is unclear.

Other relevant outcomes were included in 10 out of 11 trials [67, 69–77]. Of these ten trials, positive findings were reported in four trials: improved mental state was reported by Rivera and his team, who evaluated a supported socialisation intervention for adults with schizophrenia, other psychotic disorders or mood disorders [77]; reduced depression and social avoidance were reported by Stravynski and colleagues, who evaluated a mixture of strategies for people with social phobia and/or avoidant personality disorder [72]. Atkinson and colleagues also reported a greater quality of life when psychoeducation/social skills training was offered to people with schizophrenia [67], and fewer emergency visits were also reported for a cohort of people with schizophrenia and psychotic symptoms receiving psychoeducation/social skills training [71]. However, Solomon and her team found no differences in psychiatric symptoms or service use for participants who received consumer-led case management [70, 74], and no clinical differences were reported by Terzian and colleagues in a social network intervention for people with schizophrenia [73].

### Interventions targeting both subjective and objective social isolation

Four trials included both subjective and objective social isolation as outcomes (Table 3).

**Table 3:** Trials that included both subjective and objective social isolation as outcomes

Main authors, sample and setting	Intervention categorisation	Intervention name	Follow-up	Subjective/objective social isolation and other outcome measures	Subjective social isolation outcomes	Objective social isolation outcomes
Group-based intervention						
Castelein [86]—106 adults aged ≥ 18 with schizophrenia or related psychotic disorders 4 mental health centres in the Netherlands (secondary care setting)	Supported socialisation	Care as usual + Guided Peer Support Group (GPSG) vs. a waiting- list (WL) condition Duration: 8 months	End-of-treatment follow-up (8 months)	Subjective social isolation outcome: the Social Support List (SSL) [90] Objective social isolation outcome: Personal Network Questionnaire (PNQ) [86] Other outcomes: quality of life; screening for psychosis	Experimental group had a significantly greater increase in esteem support (*p* = 0.02), compared to WL^a^	Experimental group had a significantly greater improvement in social contacts with peers after the sessions (*p* = 0.03), compared to WL
Gelkopf [87]—34 adults with chronic schizophrenics by DSM-III-R 7 chronic schizophrenia wards in Israel (secondary care setting)	Changing cognitions	Video projection of humorous movies vs. treatment-as-usual control group Duration: 3 months	1 medium-term follow-up: 2 weeks	Subjective social isolation outcome: the Social Support Questionnaire 6 (SSQ6) [91] Objective social isolation outcomes: 2 measures of social network sum up the size and dispersion; 4 measures assess the source of the support	A significantly greater improvement in the experimental group than the control group, in perceived amount of support from staff (*F* = 7.90, *p* < 0.01), emotional support (*F* = 4.80, *p* < 0.05), and instrumental support, (*F* = 4.94, *p* < 0.05) No significant results in satisfaction towards the support (*F* = 1.90, *p* > 0.05^b^)	A significantly greater improvement in the experimental group than the control group in the number of supporters (*F* = 4.87, *p* < 0.05)
Individual-based intervention						
Ammerman [88]—93 females aged from 16–37 with MDD A community-based home visiting programme in Southwestern Ohio and Northern Kentucky in the US (general population setting)	Changiing cognitions	In-Home Cognitive Behavioural Therapy (IH-CBT) + home visiting vs. home visit alone Duration: about 5 months	End-of-treatment follow-up (5 months) 1 medium-term follow-up: 3 months	Subjective social isolation outcome: Interpersonal Support Evaluation List (ISEL) [62] Objective social isolation outcome: Social Network Index (SNI) [92] Other outcome: psychiatric symptoms	IH-CBT group reported a greater increase in social support (*p* < 0.001) than SHV. Small effect size for social support (0.38) at post-treatment, and moderate effect size (0.65) at follow-up	No significant between-group difference in network size (*F* = 1.88, *p* > 0.05), network diversity (*F* = 0.63, *p* > 0.05), and embedded networks (*F* = 2.23, *p* > 0.05)^c^
Mixed format (group- and individual-based)						
Schene [89]—222 adults aged > 60 with various mental disorders University Psychiatric Clinic of the Academic Hospital in Utrecht, the Netherlands (secondary care setting)	Psychoeducation/social skills training, and supported socialisation	Psychiatric day treatment vs. inpatient treatment (treatment-as-usual) Duration: on average 37.6 weeks for day treatment, and 24.9 weeks for inpatient treatment	End-of-treatment follow-up (on average 37.6 weeks for day treatment, 24.9 weeks for inpatient treatment) 1 medium-term follow-up: 6 months	Subjective and objective social isolation outcomes: Social Network and Social Support Questionnaire (SNSS) [93] Other outcomes: mental state; psychiatric symptoms; social dysfunction	No significant between-group difference in social support (*F* = 0.20, *p* > 0.05), and no change over time (*F* = 1.25, *p* > 0.05)^d^	No significant between-group difference in network scope (*F* = 0.05, *p* > 0.05) and network contacts (*F* = 0.02, *p* > 0.05)

^a^Effect size and confidence interval not available in the paper

^b^Effect size, confidence interval and actual *p* value not available in the paper

^c^Effect size, confidence interval and actual *p* value not available in the paper

^d^Effect size, confidence interval and the actual *p* value not available in the paper

One trial [86] only included end-of-treatment outcomes. The follow-up period was between 2 weeks and 6 months in the other three trials [87–89]. Measures with established reliability and validity were used in three trials, but the measure in one trial [86] was developed by the team and not clearly described. One trial included people with common mental health problems, two included people with severe mental illness, and one included people with a variety of different mental health diagnoses. Two trials had fewer than 100 participants and only one had more than 200. A sample size calculation was included in one trial.

One trial involved an individual intervention, two trials involved group interventions, and one trial combined individual, group and phone elements. The length of interventions ranged from 3 to 8 months. One trial was of a supported socialisation intervention, two of cognitive modification, and another used a mixture of strategies (Appendices 4 and 5).

Two trials were judged as at low risk of bias for sequence generation, two trials were at low risk for allocation concealment, and only one trial included a strategy for missing data. All trials were at high risk of bias for their blinding process and other sources of bias, but all were at low risk for selective outcome reporting (Appendix 6).

In all four trials, an intervention group was compared to either a waiting-list or a treatment-as-usual control group. Significant between-group differences in subjective social isolation were demonstrated in three out of four trials: a peer support group for adults with psychosis [86], a group-based intervention involving showing humorous movies for adults with schizophrenia [87], and in-home cognitive behavioural therapy for women with major depressive disorders [88]. Of the three trials in which a significant effect on subjective isolation was reported, significant effects on objective social isolation were also reported in two trials [86, 87]. Schene and colleagues [89] did not find any significant between-group differences for either outcome in a diagnostically mixed sample receiving psychiatric day treatment, compared to standard inpatient care.

In terms of other relevant outcomes, reduction in symptoms were reported by authors in three out of four trials: by Schene and colleagues who examined a mixture of strategies for people with a range of diagnoses [89], by Ammerman and his team who evaluated an intervention with a cognitive modification component for women with depression [88], and by Castelein and colleagues, who evaluated a supported socialisation intervention for people with schizophrenia [86]. Castelein and colleagues also reported additional benefits for quality of life.

### Overall results

Table 4 summarises the results for each type of intervention for subjective and objective social isolation, including the ones targeting both subjective and objective social isolation.

**Table 4:** Summary of different types of intervention and results: objective and subjective social isolation

Type of intervention	Comparison	Outcomes for subjective isolation	Outcomes for objective isolation
Changing cognitions	Intervention versus TAU or no treatment	2/4 studies found significant positive results	1/2 studies found significant positive results
	two or more active treatments	0/2 studies found significant positive results for one form of intervention over others	N/A
Social skills training and/or psychoeducation	Intervention versus TAU or no treatment	0/3 studies found significant positive results	1/2 studies found significant positive results
	Two or more active treatments	1/1 studies found significant positive results for one form of intervention over others	N/A
Supported socialisation	Intervention versus TAU or no treatment	1/2 studies found significant positive results	1/1 studies found significant positive results
	Two or more active treatments	0/1 studies found significant positive results for one form of intervention over others	1/1 studies found significant positive results for one form of intervention over others
Wider community approaches	Intervention versus TAU or no treatment	N/A	N/A
	Two or more active treatments	N/A	N/A
Mixed approaches (interventions with mixed components)	Intervention versus TAU or no treatment	0/5 studies found significant positive results	3/4 studies found significant positive results
	2 or more active treatments	0/1 studies found significant positive results for one form of intervention over others	0/4 studies found significant positive results for one form of intervention over others

Of all the trials that included a subjective social isolation measure (i.e. combining 15 trials including only a subjective social isolation measure and the four trials targeting both subjective and objective social isolation—19 trials in total), positive results were reported in two out of the six trials that examined interventions with a cognition modification component, one out of the three trials of supported socialisation, and one out of the four trials of social skills training/psychoeducation programmes. Authors who evaluated mixed intervention strategies found no significant positive results. None of the trials evaluated wider community approaches alone.

Regarding all the trials which included an objective social isolation measure (i.e. 15 trials), findings from one out of the two trials that involved changing cognitions, one out of the two trials that examined social skills training and psychoeducation, three out of the eight trials with a mixed intervention strategy, as well as all trials (i.e. two trials) that provided supported socialisation, suggested improvements in objective social isolation. No included trials for objective social isolation involved wider community approaches alone. Small samples and lack of sample size calculations need to be borne in mind throughout.

In many of the included trials, subjective and/or objective social isolation was one of several outcomes (with no clearly specified primary outcome), and for some trials, strategies to reduce social isolation were part of an often much broader service improvement approach (e.g. [70, 74, 76, 89]). Just six trials [43, 72, 73, 75, 86, 87] had a measure of social isolation as the clearly stated primary outcome. Four out of these six trials included either a waiting-list or a treatment-as-usual control group [73, 75, 86, 87], and findings from all of these indicated a superior effect of their intervention compared to the control condition on the trials’ objective social isolation outcomes. In these trials, one intervention involved mixed strategies for adults with schizophrenia [73]; one involved supported socialisation for adults with schizophrenia/psychosis [86]; one compared two treatment groups (i.e. systematic desensitisation and social skills training) to a waiting-list control in a sample of people with personality disorders or neurosis [75]; and another trial investigated an intervention with a cognitive modification component for adults with schizophrenia [87]. Similar to Marzillier’s trial [75], Stravynski and colleagues [72] also offered cognitive modification and social skills training to a comparable sample. Stravynksi’s trial involved a very small sample and the authors failed to find any additional improvement when a cognitive modification element was added to their social skills training. In one trial of four active conditions without a control group, for people with varied Axis I mental health diagnoses (e.g. depression, bipolar disorders) [43], the authors reported a positive effect of its psychoeducation component over other intervention groups (e.g. music alone), though only on one outcome: perceived social support from friends. In most trials in which subjective or objective social isolation was specifically targeted as the primary outcome, and interventions were tailored accordingly, positive results were reported: this specific focus may be important for intervention effectiveness.

## Discussion

With growing interest in tackling subjective and objective social isolation due to the negative health impact of both issues, we conducted the current systematic review to summarise evidence from RCTs for interventions with subjective and/or objective social isolation as main outcome(s) in people with mental health problems. Given the quality and sample size of many included studies, conclusions need to be cautious. The strategies found were extremely diverse. A tendency not to clearly specify primary outcomes in earlier trials meant that some of the trials meeting our criteria were broad socially oriented programmes in which social isolation measures were among a number of outcomes. The great diversity of interventions and low quality of reporting in some trials made meta-analysis inappropriate.

A small number of mainly small trials (in a mixture of populations) provided some evidence that perceived social support may be increased by interventions that involve cognitive modification (e.g. [88]), although there were also some trials, generally with short follow-ups in which an effect was not found (e.g. [46, 47]). Small sample sizes and lack of sample size calculations make it difficult to draw firm conclusions from the negative studies. In terms of psychoeducation/social skills training programmes (e.g. [42, 43]), no clear supporting evidence was found for subjective social isolation, although an evaluation of one educational intervention found positive results on one subscale [43]. Again, the lack of large well-powered trials with clearly focused interventions makes definitive conclusions hard to draw.

There is also evidence supporting some of the interventions targeting objective social isolation (e.g. [67–69]). However, studies included a wide range of types of intervention, none of which can be identified as clearly more effective than others. Group-based interventions and interventions involving supported socialisation appeared to have more evidence supporting their effectiveness in reducing objective social isolation than they do for subjective social isolation. All objective social isolation interventions delivered in a group format demonstrated effectiveness, compared to only two out of eight individual-based interventions, though again, lack of power and of clear theory-driven methods for alleviating isolation diminish our confidence in making firm negative conclusions. For people with mental health problems (especially people with psychosis), initiating and maintaining good social relationships can be disrupted by several difficulties, including self-stigma, psychiatric symptoms, and societal discrimination [94]. Therefore, group-based interventions may offer a pathway to initiating social contacts and practising social skills in a relatively safe environment. It is of note that a good quality multicentre trial of peer support groups from the Netherlands, in which the supported socialisation intervention led to increased social contact, did not improve subjective social isolation [8]. The supported socialisation interventions in our review did not have clear effects on subjective social isolation either. It thus seems possible that supported socialisation is more effective in reducing objective than subjective social isolation. There are two possible explanations: first, a lack of social relationships may not be the only factor contributing to subjective social isolation: social cognitions may also play a significant role [95]; second, organised groups may simply not be an effective way to help lonely people initiate meaningful friendships, start intimate relationships, or maintain or improve current relationships. However, most included studies were small and not informed by power calculations, so few definite conclusions can be drawn.

Some (e.g. [68, 69]), but not all (e.g. [70, 72]), interventions with multiple components appeared to have substantial impacts on improving objective social isolation. Solomon and her colleagues [70, 74] failed to find any significant between-group difference in their two trials, which demonstrated comparable effectiveness of consumer-provided and non-consumer provided support in terms of clinical and psychosocial outcomes. However, it must be noted that multi-component interventions often had multiple outcomes and multiple aims extending beyond alleviating social isolation: they met our inclusion criteria because social isolation was among a number of outcomes, with no specified primary outcome. Psychoeducation programmes/social skills training were evaluated in only two trials [67, 71]: only Atkinson found a significant change on their social isolation outcome, so the effectiveness of this type of intervention remains unclear. It is possible that, as suggested by Mann and colleagues [39], social skills training is more suitable for client groups who are preparing to attend wider community groups, or that it works best when combined with other types of interventions (e.g. [68]).

Cognitive modification has not been shown to be effective for objective social isolation: of the two trials using this technique to target objective social isolation [87, 88], significant changes were only observed in one trial [87] with a short follow-up period and a small sample size. In another trial [72], cognitive modification showed no additional benefits when added to social skills training, but the sample was very small and firm conclusions could not be drawn.

We did not find any relevant trial on interventions focusing on the wider community approaches alone, such as the social prescribing and community asset-development approaches described by Mann and her team [39]. It is possible that interventions where the focus is at community-level are difficult to evaluate via individually randomised trials, but such trials are potentially feasible for individual-level approaches such as social prescribing.

### Limitations

To the best of our knowledge, this systematic review is the first to provide an overall synthesis of evidence on the effectiveness of interventions for subjective and/or objective social isolation across a range of mental health diagnoses. But it has important limitations. First, we included trials in which subjective and/or objective social isolation was either a primary outcome or one of a list of outcomes with none specified as primary. This means that we have excluded some trials which might offer relevant evidence based on secondary outcomes, and we have included trials where social isolation is one of a list of outcomes, but may not have been clearly the principal target of the intervention. Few of the included trials involved theory-driven interventions for which social isolation was the clear main target. Second, the conclusions we have drawn are limited by the heterogeneity of the intervention types and patient groups, and the low methodological quality of many included trials. Each type of intervention was only evaluated in a small number of trials and the content of programmes varied greatly. Factors such as lack of information on randomisation processes and allocation concealment resulted in high ratings for risk of bias in many of the studies. Many studies were essentially feasibility or pilot trials, with small sample sizes and no underpinning power calculations: thus no clear conclusions could be drawn from either positive or negative results from these studies, including several trials comparing two or more active interventions. As expected, variations between studies regarding interventions, study participants and outcomes measurement methods precluded meta-analysis. Additionally, four trials did not include a well-established outcome measure (e.g. [45, 73]). Last, although there were no restrictions on the language of the included trials and no filter of language was used during the literature search, no eligible trials in other languages were retrieved. Great efforts were made to retrieve all relevant papers, but some trials in other languages may have been missed.

### Research implications

Compared with objective social isolation and social support, the concept of loneliness has only recently been subjected to scientific research. This review identified few trials that included loneliness as their main outcome, and none yielded positive results. Recently published pilot trials have established that loneliness is a feasible target for intervention in severe mental illness, either through face-to-face or digital programmes [31, 96]. However, there is still a pressing need to evaluate interventions for loneliness scientifically in large-scale RCTs, given growing enthusiasm for these approaches. We have thus identified an important gap in the literature.

Some trials focusing on objective social isolation and perceived social support were retrieved, but some advances need to be made to develop a substantial body of evidence in this area. First, most trials were vague in articulating a theoretical basis. The development of a clear theory of change is now regarded as an important step in the development of complex health interventions [97, 98]. Developing such theoretical models could helpfully be informed by a richer understanding of experiences of subjective and objective social isolation among people with mental health problems and their views about what may alleviate these. Thus a co-produced approach to intervention development may result in interventions with a more robust theoretical basis and a closer fit to recipients’ needs. Second, greater advances are likely to be made in this area if future trials can specify interventions in greater detail, and if future systematic reviews use clear systems, such as those applied in this review, to categorise interventions. We found that the descriptions of most interventions were typically vague, and most involved several components and delivery methods. Thus the main components of each intervention were often unclear, and exactly which elements contributed to any positive outcomes was difficult to determine. However, this should not limit the development of future interventions with multiple components (e.g. interventions combining cognitive modification with addressing social/environmental barriers to social participation and developing social relationships). Cacioppo and colleagues [99] proposed that loneliness is a multi-dimensional concept, and there is a clear distinction between intimate, relational, and collective loneliness. Thus, as a complex multi-faceted phenomenon, loneliness may well need to be addressed through multiple means.

Computer/mobile technology has become a popular format for the implementation of interventions in the medical field. Online interventions, including online support groups or chatrooms, may potentially be an effective way to provide social support [100]. However, only four trials targeting online interventions were retrieved in the current review and none has shown positive effects. Authors from existing systematic reviews [101, 102] conclude that there is great future potential for the development and utilisation of mobile apps in the mental health field. Meta-analyses have also demonstrated the use of online interventions as an acceptable and practical method to deliver healthcare for people with depression and anxiety [103, 104]. Another systematic review examined the feasibility of web- and phone-based interventions for people with psychosis: authors supported the feasibility of such interventions, and reported a range of positive outcomes in some of the studies included, including improved social connectedness and socialisation [105]. However, only few trials included in this review were RCTs and social isolation was not generally a primary outcome so that studies were not eligible for inclusion in the current review. One pilot trial has also investigated a novel online intervention called HORYZONS for young people with First Episode Psychosis (FEP), and participants became more socially connected after using HORYZONS [106]. Currently, a full trial utilising a single-blind RCT design to evaluate the effectiveness of this intervention over an 18-month follow-up period is taking place for young people with FEP [107]. In another recent feasibility trial [96], authors developed a digital smartphone application (app) named +Connect, which sought to utilise a positive psychology intervention (PPI) for young adults with early psychosis. The programme was found to be effective in reducing loneliness from baseline to 3-month post-intervention follow-up. Programme users also highlighted the benefits in their social lives of positive reinforcement provided by the app. Thus, although digital interventions have been insufficiently tested in substantial RCTs to date, it is feasible to implement such interventions for people with severe mental health problems in order to reduce loneliness, and there is a need for future research to develop and further examine digital interventions on a larger scale. Additionally, the successful implementation of interventions involving positive psychology in the two pilot trials from Lim and her colleagues [31, 96] supports the idea that subjective social isolation is increasingly recognised as a primary treatment outcome for people with psychosis in the mental health field, and future research should also focus on the development and examination of new types of intervention that target loneliness directly for people with mental health problems.

Other forms of intervention that are so far untested but with potential to have effects on loneliness and social isolation include “friends interventions”, which involve patients’ friends in treatment with the aim of strengthening relationships [108] and other interventions aimed at reinvigorating or restoring existing relationships [109]. By focusing on existing social networks, this type of intervention has potential to improve the quality of social relationships already established prior to mental health diagnosis. Beyond the individual level, there is also potential for the development and robust evaluation of the impact on people with mental health problems of interventions on a larger scale, for example, aimed at developing social connections within groups, communities or neighbourhoods, or at maximising the use of existing community assets [39]. Interventions involving wider communities have been seen as crucial in providing social opportunities for people with mental health problems to engage with their local communities and increase their sense of belonging and self-confidence [39]. Indirect interventions targeting upstream factors that contribute to social isolation [110–113] are potentially effective, such as programmes to improve housing and reduce poverty.

### Clinical implications

There is substantial evidence demonstrating the significant impact of objective and subjective social isolation on health. However, lack of empirical evidence on the efficacy of targeted interventions means that we cannot yet make clear recommendations for interventions. As argued in a recent Lancet editorial [114], there is a need for life science funding prioritising under-researched social, behavioural, and environmental determinants of health. Subjective and objective social isolation are among the social determinants of health that have received insufficient attention. Some of the research we report does provide a starting point for further work: in a few studies there is some evidence of effectiveness, while other studies with small samples have at least demonstrated that interventions are feasible and acceptable.

To conclude, based on this systematic review, current evidence does not yet clearly support scaled-up implementation of any types of intervention for subjective or objective social isolation in mental health services. Even though cognitive modification shows some promise for subjective social isolation, and interventions with mixed approaches and supported socialisation have also demonstrated their effectiveness for objective social isolation, quality of these trials limited our confidence in publicising their effectiveness. Therefore, innovation in intervention development and more high-quality research is needed. We also note that there is much innovative and interesting practice in this field that is not currently underpinned by research, especially in the voluntary sector: defining, establishing the theoretical premises for and evaluating existing models may thus be a promising direction.

## Appendix 1: Measures and scales for subjective and objective social isolation

**Table:**

	Measures	Description	For which populations
Subjective social isolation	The University of California at Los Angeles (UCLA) Loneliness Scale [115]	A unidimensional scale to assess the frequency and intensity of one’s lonely experiences, 20 items	General population (e.g. elderly, lonely students, immigrants) People with mental health problems (e.g. psychiatric inpatients, people with depression)
	UCLS-8 [6]	A short-form of UCLA Loneliness Scale, 8 items	General population (e.g. university students, adolescents, elderly sample) People with mental health problems (e.g. people with depression, mixed sample with various diagnoses)
	The De Jong-Gierveld Loneliness Scale [116]	A 11-item scale measures the feeling of severe loneliness, contains 5 positive and 6 negative items A short-form contains 6 items of the original De Jong-Gierveld Loneliness Scale (3 items for emotional loneliness and 3 items for social loneliness)	General population (e.g. national survey samples from several countries, elderly Chinese) People with mental health problems (e.g. mixed samples with various diagnoses)
	Multidimensional Scale of Perceived Social Support (MSPSS) [57]	A 12-item scale to measure perceived overall amount of social support and support from significant other/friends/family	General population (e.g. Chinese university students, young adults, adults with physical disabilities) People with mental health problems (e.g. people with post-traumatic stress disorder, women with severe depressive symptoms)
Objective social isolation	Social Network Index (SNI) [92]	A 12-item scale, measures the number of people one has regular contact with	General population (e.g. women with breast cancer, people with severe traumatic brain injury, African-Americans in urban area) People with mental health problems (e.g. old adults with depressive symptoms, people with post-traumatic stress disorder)
	The Pattison Psychosocial Kinship Inventory (PPKI) [117]	Measures the number of people and relationships one considers as important	General population (e.g. dysfunctional families) People with mental health problems (e.g. adults with schizophrenia, people with psychosis)
Measures focus on both domains	Lubben Social Network Scale (LSNS-6)	A revised version, contains 6 items, evaluates the quantity and quality of one’s relationship with family and friends	General population (e.g. community-dwelling elderly, Korean American caregivers) People with mental health problems (e.g. mixed samples with different diagnoses, depressed immigrants)
	Social Network Schedule (SNS) [78]	A 6-item scale, measures both quantitative (i.e. the size of one’s social network, the frequency of social communication and the time one spent on socialisation) and qualitative (i.e. quality and intimacy of one’s social relationships, the intensity of social interactions) aspects of one’s social connections	People with mental health problems (e.g. people with non-organic psychosis, people with intellectual disability)
	Medical Outcomes Study (MOS) Social Support Scale [64]	A 19-item survey measures dimensions of social support: emotional/informational, tangible, affectionate and positive social interactions	General population (people with heart failure in Hong Kong, mothers with children in treatment) People with mental health problems (e.g. adults with schizophrenia spectrum or affective disorder)
	Interview Schedule for Social interaction (ISSI) [61]	50 items, measures the availability and perceived adequacy of attachment and social integration	General population (e.g. patients with rheumatoid arthritis, people from Canberra suburbs) People with mental health problems (e.g. outpatients with schizophrenia, inpatient male offenders)

## Appendix 2: Existing systematic reviews and meta-analyses

**Table:**

Authors (published years)	Published years of included studies	Review method	Included participants	How interventions were categorised	Number of studies	Types of study included
Subjective social isolation interventions						
Findlay [30]	1982–2002	Systematic review	Older people	(1) Increase social support (2) Psychoeducation/social skills training	17	RCTs, non-randomised comparison studies
Cattan et al. [32]	1970–2002	Systematic review	Older people	(1) Social skills training (2) Provide social support (3) Psychoeducation/social skills training	30	RCTs, non-randomised comparison studies
Dickens et al. [33]	1976–2009	Systematic review	Older people	(1) Increase social opportunities (2) Provide social support (3) Psychoeducation/social skills training (4) Address maladaptive social cognitions	32	RCTs, non-randomised comparison studies
Masi et al. [34]	1970–2009	Meta-analysis	Adults, adolescents and children	(1) Increase social opportunities (2) Provide social support (3) Address maladaptive social cognitions (4) Provide social skill trainings	50	RCTs, non-randomised comparison studies
Perese and Wolf [35]	Unclear	Narrative synthesis	People with mental health problems	Social network interventions: include support groups, psychosocial clubs, self-help groups, mutual help groups and volunteer groups	36	Unclear
Objective social isolation interventions						
Newlin et al. [36]	Up to September 2014	Systematic Review and modified narrative synthesis	People with mental health problems	All types of psychosocial interventions	16	RCTs, non-randomised comparison studies and qualitative studies
Anderson et al. [37]	2008–2014	Systematic review	People with psychosis	All types of social network interventions	5	RCTs
Webber and Fendt-Newlin [38]	2002–2016	Narrative synthesis	People with mental health problems	Social participation interventions: include social skills training, supported community engagement, group-based community activities, employment interventions and peer support interventions	19	RCTs, non-randomised comparison studies, and qualitative studies

## Appendix 3: Search terms in Medline and PsycINFO

Same terms were used for the search in Web of Science with minor changes.

**Table:**

#	Search term
1	loneliness.mp. [mp = title, abstract, original title, name of substance word, subject heading word, keyword heading word, protocol supplementary concept word, rare disease supplementary concept word, unique identifier]
2	Loneliness.mp. or Loneliness/
3	lonely.mp. [mp = title, abstract, original title, name of substance word, subject heading word, keyword heading word, protocol supplementary concept word, rare disease supplementary concept word, unique identifier]
4	(social support adj5 (subjective or personal or perceived or quality)).mp. [mp = title, abstract, original title, name of substance word, subject heading word, keyword heading word, protocol supplementary concept word, rare disease supplementary concept word, unique identifier]
5	Confiding relationship*.mp. [mp = title, abstract, original title, name of substance word, subject heading word, keyword heading word, protocol supplementary concept word, rare disease supplementary concept word, unique identifier]
6	Social isolation.mp. or Social Isolation/
7	Social network*.mp. [mp = title, abstract, original title, name of substance word, subject heading word, keyword heading word, protocol supplementary concept word, rare disease supplementary concept word, unique identifier]
8	socially isolated.mp. [mp = title, abstract, original title, name of substance word, subject heading word, keyword heading word, protocol supplementary concept word, rare disease supplementary concept word, unique identifier]
9	1 or 2 or 3 or 4 or 5 or 6 or 7 or 8
10	Mental Disorders/
11	Alcoholism/or Middle Aged/or Child Behaviour Disorders/or Child/or Adolescent/or Stress Disorders, Post-Traumatic/or Adult/or Depression/or Mental Disorders/or mental health problems.mp. or Substance-Related Disorders/
12	Bipolar Disorder/or Psychotic Disorders/or Aged/or Stress, Psychological/or Middle Aged/or Community Mental Health Services/or Adult/or Mental Disorders/or mental illnesses.mp. or Schizophrenia/
13	mental.mp. [mp = title, abstract, original title, name of substance word, subject heading word, keyword heading word, protocol supplementary concept word, rare disease supplementary concept word, unique identifier]
14	Psychiatr*.mp. [mp = title, abstract, original title, name of substance word, subject heading word, keyword heading word, protocol supplementary concept word, rare disease supplementary concept word, unique identifier]
15	Schizo*.mp. [mp = title, abstract, original title, name of substance word, subject heading word, keyword heading word, protocol supplementary concept word, rare disease supplementary concept word, unique identifier]
16	Psychosis.mp. [mp = title, abstract, original title, name of substance word, subject heading word, keyword heading word, protocol supplementary concept word, rare disease supplementary concept word, unique identifier]
17	Depress*.mp. [mp = title, abstract, original title, name of substance word, subject heading word, keyword heading word, protocol supplementary concept word, rare disease supplementary concept word, unique identifier]
18	Suicid*.mp. [mp = title, abstract, original title, name of substance word, subject heading word, keyword heading word, protocol supplementary concept word, rare disease supplementary concept word, unique identifier]
19	Mania*.mp. [mp = title, abstract, original title, name of substance word, subject heading word, keyword heading word, protocol supplementary concept word, rare disease supplementary concept word, unique identifier]
20	Manic.mp. [mp = title, abstract, original title, name of substance word, subject heading word, keyword heading word, protocol supplementary concept word, rare disease supplementary concept word, unique identifier]
21	Bipolar.mp. [mp = title, abstract, original title, name of substance word, subject heading word, keyword heading word, protocol supplementary concept word, rare disease supplementary concept word, unique identifier]
22	Anxiety.mp. [mp = title, abstract, original title, name of substance word, subject heading word, keyword heading word, protocol supplementary concept word, rare disease supplementary concept word, unique identifier]
23	Personality disorder*.mp. [mp = title, abstract, original title, name of substance word, subject heading word, keyword heading word, protocol supplementary concept word, rare disease supplementary concept word, unique identifier]
24	Eating disorder*.mp. [mp = title, abstract, original title, name of substance word, subject heading word, keyword heading word, protocol supplementary concept word, rare disease supplementary concept word, unique identifier]
25	Anorexia.mp. [mp = title, abstract, original title, name of substance word, subject heading word, keyword heading word, protocol supplementary concept word, rare disease supplementary concept word, unique identifier]
26	Bulimia.mp. [mp = title, abstract, original title, name of substance word, subject heading word, keyword heading word, protocol supplementary concept word, rare disease supplementary concept word, unique identifier]
27	PTSD.mp. [mp = title, abstract, original title, name of substance word, subject heading word, keyword heading word, protocol supplementary concept word, rare disease supplementary concept word, unique identifier]
28	Post-traumatic stress disorder*.mp. [mp = title, abstract, original title, name of substance word, subject heading word, keyword heading word, protocol supplementary concept word, rare disease supplementary concept word, unique identifier]
29	10 or 11 or 12 or 13 or 14 or 15 or 16 or 17 or 18 or 19 or 20 or 21 or 22 or 23 or 24 or 25 or 26 or 27 or 28
30	9 and 29
31	Clinical trial.mp. [mp = title, abstract, original title, name of substance word, subject heading word, keyword heading word, protocol supplementary concept word, rare disease supplementary concept word, unique identifier]
32	Controlled study.mp. [mp = title, abstract, original title, name of substance word, subject heading word, keyword heading word, protocol supplementary concept word, rare disease supplementary concept word, unique identifier]
33	Randomized controlled trial.mp. [mp = title, abstract, original title, name of substance word, subject heading word, keyword heading word, protocol supplementary concept word, rare disease supplementary concept word, unique identifier]
34	Randomised controlled trial.mp. [mp = title, abstract, original title, name of substance word, subject heading word, keyword heading word, protocol supplementary concept word, rare disease supplementary concept word, unique identifier]
35	RCT.mp. [mp = title, abstract, original title, name of substance word, subject heading word, keyword heading word, protocol supplementary concept word, rare disease supplementary concept word, unique identifier]
36	31 or 32 or 33 or 34 or 35
37	30 and 36

## Appendix 4: Characteristics of included trials

**Table:**

Main author	Setting	Participants	Follow-up	Social isolation outcomes	Other outcomes	Intervention type
Subjective social isolation trials						
Kaplan [53]	Online intervention, US	300 adults with a diagnosis of a schizophrenia spectrum or an affective disorder	2 medium-term follow-ups: 4 and 12 months (post-baseline)	The Medical Outcomes Study (MOS) Social Support Survey [64]	(1) Personal recovery (2) Quality of Life (3) Psychiatric symptoms	Supported socialisation
Hasson-Ohayon [42]	Psychiatric community rehabilitation centre, Israel	210 adults with severe mental illness	End-of-treatment follow-up	Multidimensional Scale of Perceived Social Support [57]	Personal recovery	Psychoeducation/social skills training
Rotondi [54]	In- and outpatient psychiatric care units and psychiatric rehabilitation centres, Pittsburgh, Pennsylvania	30 patients aged ≥ 14 with schizophrenia or schizoaffective disorder	2 medium-term follow-ups: 3 and 6 months (post-baseline)	The informational support and emotional support subscales of the instrument that was developed by Krause and Markides [65]	N/A	Psychoeducation
Silverman [43]	Acute care psychiatric unit, a university hospital, the Midwestern region, US	96 adults with varied Axis I diagnoses	End-of-treatment follow-up	The Multidimensional Scale of Perceived Social Support (MSPSS) [57]	N/A	Psychoeducation
Boevink [44]	Mental health care organisations, the Netherlands	163 adults with varied mental illness	1 medium-term follow-up: 12 months (post-baseline) One long-term follow-up: 24 months (post-baseline)	The De Jong-Gierveld Loneliness Scale [58]	(1) Quality of Life (2) Psychiatric symptoms	Supported socialisation
Zang [46]	Beichuan County, China	30 aged 28–80 with PTSD	End-of-treatment follow-up 2 medium-term follow-ups: 1 week or 2 weeks, and 3 months	The Multidimensional Scale of Perceived Social Support (MSPSS) [57]	(1) Anxiety and depressive symptoms (2) PTSD symptoms	Changing cognitions
Zang [47]	Beichuan County, China	22 aged 37–75 with PTSD	End-of-treatment follow-up 2 medium-term follow-ups: 2 weeks and 2 months	The Multidimensional Scale of Perceived Social Support (MSPSS) [57]	(1) Subjective level of distress (2) Depressive symptoms	Changing cognitions
Gawrysiak [48]	A public Southeastern university, US	30 aged ≥ 18 with depression	1 medium-term follow-up: 2 weeks	The Multidimensional Scale of Perceived Social Support (MSPSS) [57]	(1) Depressive symptoms (2) Anxiety symptoms	Psychoeducation/social skills training and supported socialisation
Bjorkman [50]	Case management service, Sweden	77 adults aged 19–51 with severe mental illness	2 long-term follow-ups: 18 and 36 months	The abbreviated version of the Interview Schedule for Social Interaction (ISSI) [61]	(1) Psychiatric symptoms (2) Quality of life	Social skills training
Mendelson [51]	Baltimore City, US	78 depressed women aged 14–41 who either were pregnant or had a child less than 6 months old	End-of-treatment follow-up 2 medium-term follow-ups: 3 and 6 months	The Interpersonal Support Evaluation List (ISEL) [62]	N/A	Changing cognitions
O’Mahen [55]	Online intervention, UK	83 women aged > 18 with MDD	End-of-treatment follow-up 1 medium-term follow-up: 6 months	The Social Provision Scale [66]	(1) Depressive symptoms (2) Anxiety symptoms	Psychoeducation and supported socialisation
Conoley [49]	Psychology Department, US	57 female psychology undergraduate students with moderate depression	End-of-treatment follow-up 1 medium-term follow-up: 2 weeks	The Revised UCLA Loneliness Scale [59] The Causal Dimension Scale [60]	Depressive symptoms	Changing cognitions
Eggert [45]	5 urban high schools, US	105 high school students with poor grades (moderate or severe depression)	2 medium-term follow-ups: 5 and 10 months (post-baseline)	Perceived social support: measured by calculating average ratings across six network support sources. The instrumental and expressive support provided by each support source was also rated	Depressive symptoms	Supported socialisation, social skills training, and wider community approaches
Masia-Warner [52]	Two parochial high schools, New York city, US	35 high school students with social anxiety disorder	End-of-treatment follow-up 1 medium-term follow-up: 9 months	Loneliness Scale [63]	(1) Anxiety symptoms (2) Social phobic symptoms (3) Depressive symptoms	Psychoeducation/social skills training, supported socialisation and changing cognitions
Interian [56]	Online intervention, US	103 veterans with PTSD	1 medium-term follow-up: 2 months (post-baseline)	The family subscale of the Multidimensional Scale for Perceived Social Support [57]	N/A	Psychoeducation and changing cognitions
Objective social isolation trials						
Solomon [70]	A community mental health centre, US	96 adults with schizophrenia or major affective disorders	2 medium-term follow-ups: 1 month and 1 year (post-baseline)	(1) Family and social contacts (2) Pattison’s Social Network scale [81]	(1) Use of services (2) Quality of Life (3) Psychiatric symptoms	Supported socialisation and wider community approaches
Aberg-Wistedt [71]	The Kungsholmen sector, Stockholm, Sweden	40 adults with schizophrenia or long-term psychotic disorder, diagnosed by DSM-III-R schizophrenic disorders	1 long-term follow-up: 2 years (post-baseline)	The number of people in participants’ social life was measured by a standardised procedure developed from work with child psychiatric patients [82]	(1) Quality of life (2) Service use	Psychoeducation/social skills training
Stravynski [72]	Maudsley Hospital, London, UK	22 adults aged 22–57 with diffuse social phobia and/or avoidant personality disorder	End-of-treatment follow-up 1 medium-term follow-up: 6 months	Structured and Scaled Interview to Assess Maladjustment (SSIAM) [83]	Depressive symptoms	Social skills training and changing cognitions
Atkinson [67]	Community clinic, South Glasgow, UK	146 registered patients with schizophrenia	End-of-treatment follow-up 1 medium-term follow-up: 3 months	A modified Social Network Schedule (SNS) [78]	(1) Quality of life (2) Psychiatric symptoms (3) Overall functioning	Psychoeducation
Terzian [73]	47 community mental health services (SPT), Italy	357 adults aged < 45 diagnosed as schizophrenia spectrum disorder by the ICD-10th	1 medium-term follow-up: 1 year (post-baseline) 1 long-term follow-up: 2 years (post-baseline)	Social network: different parameters of relationships were assessed, all were summarized into a score	(1) Psychiatric symptoms (2) Hospitalisation over the follow-up year	Supported socialisation and wider community approaches
Hasson-Ohayon [68]	3 psychiatric rehabilitation agencies and the University Community Clinic, Bar-Ilan University, Israel	55 adults aged 21–62 with various serious mental illness	1 medium-term follow-up: 6 months	Social Functioning Scale (SFS) [79]	N/A	Wider community approaches, psychoeducation/social skills training and changing cognitions
Rivera [77]	A city hospital, New York, US	203 adults with a psychotic or mood disorder on axis I	2 medium-term follow-ups: 6 and 12 months (post-baseline)	A modification of the Pattison Network Inventory [85]	(1) Quality of life (2) Psychiatric symptoms	Supported socialisation
Solomon [74]	A community mental health centre, US	96 adults with schizophrenia or major affective disorders	2 medium-term follow-ups: 1 month and 1 year (post-baseline) 1 long-term follow-up: 2 years (post-baseline)	Pattison’s Social Network [81]	(1) Quality of Life (2) Psychiatric symptoms	Supported socialisation and wider community approaches
Marzillier [75]	The Maudsley Hospital, UK	21 adults aged 17–43 with a diagnosis of personality disorder or neurosis	End of treatment follow-up 1 medium-term follow-up: 6 months	Revised-Social Diary and Standardised Interview Schedule [75]	(1) Anxiety disorders (2) Mental state (3) Personality assessment	Social skills training and changing cognitions
Bøen [69]	2 municipal districts, eastern and western Oslo, Norway	138 seniors with mild depression	End-of-treatment follow-up	The Oslo-3 Social Support Scale [80]	(1) Depressive symptoms (2) Life satisfaction	Supported socialisation and wider community approaches
Cole [76]	St. Mary’s hospital, Montreal, Canada	32 adults with major depression, dysthymic disorder or other affective disorder	3 medium-term follow-ups: 4, 8 and 12 weeks (post-baseline)	The Older Americans Research and Service Centre Instrument (OARS) [84]	(1) Mental state (2) Symptoms	N/A
Trials for both subjective and objective social isolation						
Schene [89]	University Psychiatric Clinic of the Academic Hospital, Utrecht, the Netherlands	222 adults aged > 60 with various mental disorders	End-of-treatment follow-up 1 medium-term follow-up: 6 months	Subjective social isolation outcome: Social Network and Social Support Questionnaire (SNSS) [93] Objective social isolation outcome: Social Network and Social Support questionnaire (SNSS) [93]	(1) Mental state (2) Psychiatric symptoms (3) Social dysfunction	Psychoeducation/social skills training, and supported socialisation
Castelein [86]	4 mental health centres, the Netherlands	106 adults aged ≥ 18 with schizophrenia or related psychotic disorders	End-of-treatment follow-up	Subjective social isolation outcome: The Social Support List (SSL) Objective social isolation outcome: Personal Network Questionnaire (PNQ) [86]	(1) Quality of Life (2) Screening for psychosis	Supported socialisation
Gelkopf [87]	7 chronic schizophrenic wards, Israel	34 adults with a diagnosis of chronic schizophrenia, based on DSM-III-R	1 medium-term follow-up: 2 weeks	Subjective social isolation outcome: The Social Support Questionnaire 6 (SSQ6) [91] Objective social isolation outcomes: (1) 2 measures of social network sum up the size and dispersion (2) 4 measures assess the source of the support	N/A	Changing cognitions
Ammerman [88]	Southwestern Ohio and Northern Kentucky, US	93 females aged from 16 to 37 with MDD	End-of-treatment follow-up 1 medium-term follow-up: 3 months	Subjective social isolation outcome: Interpersonal Support Evaluation List (ISEL) [62] Objective social isolation outcome: Social Network Index (SNI) [92]	Psychiatric symptoms	Changing cognitions

## Appendix 5: Characteristics of interventions

**Table:**

Main author	Intervention and control group	Mode of delivery	Number of sessions + duration of each session + duration of intervention	Intervention descriptions	Characteristics of intervention providers
Subjective social isolation					
Kaplan [53]	Experimental peer support listserv vs. experimental peer support bulletin board vs. waiting-list control group	Online	Unclear, overall duration of the study was 12 months	Experimental peer support listserv: participants communicated anonymously with each other via a group distribution email list Experimental peer support bulletin board: participants were instructed on how to create account and log in to board	The online communication of both listserv and bulletin board group were solely peer directed, but technical support was provided via phone or email
Hasson-Ohayon [42]	Illness Management and Recovery Programme vs. treatment-as-usual	Face-to-face sessions (group)	Weekly sessions, an hour each session Duration of the intervention was 8 months	Intervention group: Illness Management and Recovery Programme is a standarised curriculum-based programme, which provides essential information and skills to people with severe mental illness. The information and skills provided are designed to help patients manage their illness and work towards their personal recovery goals. In this study, educational handouts in Hebrew were provided to participants, focused primarily on self-management, personal goals, social support, medication use, relapse prevention, and coping with psychiatric symptoms	Interventions were led by two clinicians, one of whom had weekly training sessions. For the first 8 months of intervention, clinicians attended monthly supervision sessions
Rotondi [54]	Telehealth intervention vs. usual care group	Online	Unclear	Intervention group: including online therapy groups, ask questions and receive answers, a library of previous questions, activities in the community, news items, and educational reading materials	The 3 therapy groups were facilitated by master of social work and PhD clinicians, they were all trained in the monitoring and management of web-based interventions
Silverman [43]	Live educational music therapy (Condition A) vs. recorded educational music therapy (Condition B) vs. education without music (Condition C) vs. recreational music therapy without education (Condition D)	Face-to-face sessions (group)	24 weekly sessions, 45 min per session Duration of intervention: 24 weeks	Condition A: live music, a scripted educational lyric analysis session using song lyrics that focused on social support Condition B: recorded music, a scripted educational lyric analysis session about lyrics that focused on social support Condition C: Without music, a scripted educational session without music concerning support and coping Condition D: investigator led the group in playing rock and roll bingo, no scripted educational session	A certified music therapist with more than 12 years of clinical psychiatric experience conducted therapy sessions
Boevink [44]	TREE + CAU vs. CAU (waiting-list control)	Face-to-face sessions (group)	The early starters: each session lasted 2 h, met every two weeks Duration of the intervention: 104 weeks The Late starters: each session lasted 2 h, met every two weeks; Duration of the intervention: 52 weeks	TREE model: (1) Training course ‘start with recovery’ (2) Developing strength (3) A one-day recovery training course	The recovery self-help working groups were facilitated by two senior peer workers, and two mental health care managers facilitated the training course
Zang [46]	NET vs. NET-R vs. waiting-list control	Face-to-face sessions (individual)	NET group: ≥ 4 sessions, 60–90 min per session, twice weekly Duration of intervention: 2 weeks NET-R group: ≥ 3 sessions, 60–120 min per session, and each session was 1–2 days apart; Duration of intervention: 1 week	For both groups, the narrative was recorded and corrected in subsequent reading sessions NET group: created a detailed biography that focused on traumatic experiences NET-R group: a modified version of NET; the participants first constructed an earthquake narrative and then an autobiography	All treatments were carried out by the first author and one female psychological counsellor; they both speak Chinese and have the Chinese national psychological counsellor certificate (master) and also were trained in the use of NET and NET-R Weekly case and personal supervisions were conducted; the counsellors were also supervised before they have contact with participants
Zang [47]	NET vs. waiting-list control group	Face-to-face sessions (individual)	NET group: 4 sessions, 60–90 min per session Duration of intervention: 2 weeks	NET group: created a chronological report of biography with a focus on traumatic experiences. A written report of their biography was provided in the last session	The team was led by the first author, consisted of 3 female therapists, and they all speak Chinese, and all have the Chinese national psychological counsellor certificate (Master) Therapists were trained for NET and they were tutored under supervision before they work with participants. Weekly case and personal supervisions were also carried out
Gawrysiak [48]	BATD vs. no treatment control	Face-to-face session (individual)	Single session lasted 90 min	BA intervention: education, assessments of values and goals, construct an activity hierarchy, selection of value-based behaviours, establish structured behavioural goals, and behavioural checkout form	One male doctoral students in clinical psychology was trained in BATD and conducted the individualised interview
Bjorkman [50]	The case management service vs. standard care	Face-to-face sessions (individual)	1.45 per week during the first 18 months, and the case manager spent on average 1.9 h in client contacts every week Duration of intervention: unclear	The case management service: moderately focused on skills training, strong emphasis on consumer input	All staff had experiences in working in social services, psychiatric services or vocational rehabilitation. The team consisted of two registered nurses and two social workers. Supervision was done by a psychiatrist and a psychologist
Mendelson [51]	Standard home visiting services + MB course vs. standard home visiting services + information on perinatal depression	Face-to-face sessions (group and individual)	6 weekly sessions, 2 h each session Duration of intervention: 6 weeks	Intervention group: Sessions cover core cognitive behavioural concepts, including pleasant activities, thoughts, and contact with others	A licensed clinical social worker or clinical psychologist
O’Mahen [55]	NetmumsHWD vs. treatment-as-usual	Online and telephone support	12-session treatment online course, weekly telephone support sessions of 20–30 min Duration of each session and intervention: unclear	NetmumsHWD: including a core behavioural activation (BA) model, a relapse prevention session, plus two optional modules. Also a chat room that was moderated by peer supporters, and weekly supported phone call from mental health workers	Mental health supporters with undergraduate degrees and 1 year of clinical qualification in psychological therapies Peer supporters had previous training in low-intensity BA, received 5 days of training in high-intensity perinatal-specific BA approach
Conoley [49]	Reframing vs. self-control vs. waiting list	Face-to-face sessions (individual)	2 sessions with 1 week apart, each session 30 min Duration of intervention: 2 weeks	Intervention groups: aimed to increase understanding in loneliness. First half of the session consisted of loneliness and reflective responses, the second half included either 3–5 positive reframing directives for reframing subjects, and self-control directives for self-control subjects	Two male doctoral students with 3-year counselling experience, received training in both interventions
Eggert [45]	PGCI vs. PGCII vs. an assessment protocol-only	Face-to-face sessions (group)	PGCI: met daily, 55 min per meeting Duration of intervention: 5 months or 90 class days in length PGCII: met daily, 55 min per meeting Duration of intervention: 10 months or 180 class days in length	Both PGCI and PGCII: small group work focused on social support; weekly monitoring of activities; and life skills training PGCI: emphasised bonding to PGC group, included training to give and receive social support; focused on motivating to change and acquire essential skills, and rehearsing real-life issues in the group setting with a main focus on problems with friends, teachers and parents PGCII: emphasised broader school bonding, included training to transfer skills to real life situations, providing and seeking social support, and developing health-promoting social activities to reduce the negative impacts of suicidal thoughts and behaviours, anger and/or depression, and drug involvement	The interventions were delivered by trained school staff who functioned as group leaders
Masia-Warner [52]	Skills for Social and Academic Success vs. waiting-list group	Face-to-face sessions (group and individual)	12 weekly group school sessions (40 min); 2 brief individual meetings (15 min); 2 monthly group booster sessions; and 4 weekend social events (90 min) Duration of intervention: 3 months	12 group sessions: 1 psychoeducational session, 1 realistic thinking session, 4 social skills training sessions, 5 exposure sessions, and 1 relapse prevention session Individual meetings: met with group leaders at least twice, aim to identify individual treatment goals and problem solving Social events: met and practiced programme skills with peers in their community	A behaviourally trained clinical psychologist and a clinical psychology graduate student co-led all groups Peer assistants: nominated by teachers and administrators, help with exposures and skill practice
Interian [56]	The Family of Heroes intervention vs. control group	Online	1 h online intervention Duration of intervention: unclear	The Family of Heroes Intervention: provided psychoeducation and stimulated conversations regarding post-deployment stress and mental health treatment; and three conversation scenarios	N/A
Objective social isolation trials					
Solomon [70]	Consumer management team vs. non-consumer management team	Face-to-face sessions (individual)	Unclear	Both consumer and non-consumer management team followed an assertive community treatment model (1) Provided activities: housing, rehabilitation and social activities (2) Case managers provided assistance and supported clients, supervised by consumer supervisor	Requirements for consumer management team: have major mental health problems, ≥ 1 previous psychiatric hospitalisation, a minimum of 14 days of psychiatric hospitalisation, or at least 5 psychiatric emergency service contacts within a year Requirements for non-consumer case management team: consisted of mental health professionals and recent college graduates
Aberg-Wistedt [71]	The intensive case management programme vs. standard services	Face-to-face sessions (individual)	1 h individual meeting every other week; psychiatric nurse/nurse assistant met with patients at least 4 h per week. Crisis intervention services were available 24 h every day and 7 days a week. Duration of intervention: 2 years	Intervention group: (1) The team provided assertive outreach; patients received skill training and instruction in critical life task (2) Specific services also provided based on individual needs and assessments (3) Family psychoeducation and support	The team consisted of a psychologist/psychiatrist, a psychiatric social worker, a social service officer, and a psychiatric nurse/nurse assistant
Stravynski [72]	Social skills training vs. Social skill training + cognitive modification	Face-to-face sessions (individual)	12 sessions, 90 min per session Duration of intervention: 14 weeks	Social skills training: focused on individual needs by discussing specific social targets; techniques included instructions, modelling, role-rehearsal, feedback, self-monitoring, and homework Social skill training + cognitive modification: previously described elements for social skills training. For cognitive modification, participants analysed a distressing event in five steps: (1) activating event with descriptions; (2) irrational beliefs; (3) emotional consequences; (4) dispute; (5) plan for new actions	Provided by one psychiatrist
Atkinson [67]	The education group vs. waiting-list control	Face-to-face sessions (group)	1.5 h per session Duration of intervention: 20 weeks	The education group: sessions generally covered schizophrenia topics, and alternated between an information session and a problem-solving session	Led by community psychiatric nurses, occupational therapists and registrars. Trainings were also provided
Terzian [73]	Social network intervention + usual treatments vs. usual treatments	Face-to-face (individual)	Unclear information regarding intervention sessions Duration of intervention: 3–6 months	Social network intervention: participants were helped to identify their possible areas of interest, and social activities were suggested	Provided by a staff member or natural facilitators such as families, neighbours, or volunteers
Hasson-Ohayon [68]	Social Cognition and Interaction Training (SCIT) + social mentoring vs. social mentoring only	Face-to-face sessions (group)	SCIT intervention: 1 h weekly session Social mentoring service: 3 weekly meetings Duration of intervention: unclear	Participants received social, leisure, support, and employment services, as well as standard services SCIT intervention group: besides intervention, they also received educational handouts, videos, and slides All received the same social mentoring services to support practical steps toward achieving personally meaningful goals	Social mentors were staff of psychiatric rehabilitation agencies Lead clinicians received training and ongoing supervision. All clinicians had experiences in providing psychiatric rehabilitation services and completed a SCIT workshop
Rivera [77]	Peer-assisted care vs. Nonconsumer assisted vs. standard care vs. clinic-based care	Face-to-face sessions (group & individual), and phone calls	Unclear information regarding intervention sessions and duration But telephone coverage is 24 h	Peer assisted care group: professionals provided conventional crisis management, therapeutic services and concrete services; paraprofessional consumers facilitated social networks and provided social support through activities, home visits and phone calls Clinic based care group: only provided office-based services	All professionals were licensed clinical social workers, also received training and supervisions Consumers had a history of multiple hospitalisations for mood or psychotic disorders, were eligible for disability benefits, relied on medication, but had 3–8 years of sobriety and stability. They had the same trainings as professional, and were supervised by social worker
Solomon [74]	Consumer case management team vs. nonconsumer management team	Face-to-face sessions (individual)	The consumer team: Three times per week The nonconsumer team: met biweekly Duration of the intervention: 2 years	Case managers offered individualised social support for community living, activities included goals related to income, living situation, social and family relations, and psychiatric treatment	Requirements for consumer case managers: have a major mental health disorder; at least one prior psychiatric hospitalisation and a minimum of 14 days of psychiatric hospitalisation, or at least 5 psychiatric emergency service contacts over a 1-year period; regular contact in community mental health services, psychosocial services, or other outpatient treatment Consumer team: 3 consumer managers and 1 nonconsumer case manager initially, later, the nonconsumer member was replaced by a consumer, and a clinical director and a psychiatrist started involved. Consumer mangers received supervisions and support Nonconsumer team: all nonconsumer managers, two specialists started involved at the second year. Managers received supervisions and support The interviewer: a trained professional research worker independent of service providers. Intensive, experiential training was provided in both the Brief Psychiatric Rating Scale (BPRS) and Addiction Severity Index (ASI)
Marzillier [75]	Systematic Desensitisation (SD) vs. Social Skills Training (SST) vs. waiting-list control	Face-to-face sessions (individual)	15 45-min sessions, once a week, occasionally twice a week Duration of intervention: 3 and half months	Systematic desensitisation: included relaxation training and hierarchy construction, practice in both imagination and reality Social skills training: combined elements of both assertive and social skills training, included role playing, modelling, and practice in real-life and with volunteers	Assessments were done by 2 independent assessors; one was a trained psychologist, and the other was a senior psychiatrist The therapist was a trained clinical psychologist with experience in behavioural treatments
Bøen [69]	A preventive senior centre group programme vs. control	Face-to-face sessions (group)	Weekly group meetings, 3 h per meeting, about 35–38 times totally; Duration of intervention: 1 year	The experimental group: included group meeting, physical training programme, and a self-help group. Transportation and warm meals were also provided	The team consisted of volunteers; all completed a training course and were supervised by a registered nurse and an experienced senior centre leader
Cole [76]	Home assessment group vs. clinic assessment group	Face-to-face sessions (individual)	Unclear	Unclear	Study psychiatrists (MC or DR) assessed participants
Trials for both subjective and objective social isolation					
Schene [89]	Psychiatric day treatment vs. inpatient treatment	Varied: mostly face-to-face sessions or phone interview (group and individual)	Day treatment: length of programme varied Average duration of intervention: 37.6 weeks Inpatient treatment: length of programmes varied Average duration of intervention: 24.9 weeks	Nine main groups of treatment programmes: (1) individual psychotherapy or supportive therapy; (2) individual counselling; (3) group psychotherapy; (4) sociotherapy; (5) family counselling; (6) occupational therapy; (7) psychomotor therapy; (8) drama therapy; (9) secondary environmental activities Extra care for day clinic participants after office hours, such as phone call or face-to-face talks with resident on duty in the clinic, or use of clinical bed	Social psychiatric nurses, psychiatrists, and psychologists
Castelein [86]	Care as usual + GPSG vs. a waiting- list condition	Face-to-face sessions (group)	90 min per session, 16 biweekly sessions Duration of intervention: 8 months	Peer support group: included about 10 patients, patients decided the topic of each session, discussing daily life experiences in pairs and groups	Nurses guided the peer groups with minimal involvement
Gelkopf [87]	Video projection of humorous movies vs. control group	Face-to-face sessions (group)	The experimental group: four times daily (5 days a week) Duration of intervention: 3 months	The experimental group: exposed exclusively to comedies The control group: 15% of the films were comedies; others are different types of films	A psychology student was involved to answer questions during experimental testing
Ammerman [88]	IH-CBT + home visiting vs. home visit alone	Face-to-face sessions (individual)	15 weekly sessions, 60 min per session with a booster session 1 month after treatment Duration of intervention: about 5 months	IH-CBT: primarily targeted depression reduction, consisted of behavioural activation, identification of automatic thoughts and schemas, thought restructuration, and relapse prevention	2 licensed master level social workers, received weekly supervision, a review of audiotaped sessions and a self-report checklist

## Appendix 6: Quality assessment

**Table:**

First author (publication year)	Sequence generation	Allocation concealment	Blinding	Incomplete outcome data	Selective outcome reporting	Other sources of bias
Kaplan [53]	Low risk	Unclear	High risk	Unclear	Low risk	Low risk
Hasson-Ohayon [42]	Low risk	Unclear	High risk	Unclear	Low risk	High risk
Rotondi [54]	Unclear	Unclear	High risk	Unclear	Low risk	High risk
Silverman [43]	Unclear	Unclear	High risk	Unclear	Low risk	Low risk
Boevink [44]	Low risk	Unclear	High risk	Unclear	Low risk	Low risk
Zang [46]	Low risk	Unclear	High risk	Unclear	Low risk	High risk
Zang [47]	Low risk	Unclear	High risk	Unclear	Low risk	High risk
Gawrysiak [48]	Unclear	Unclear	High risk	Unclear	Low risk	Low risk
Bjorkman [50]	Low risk	Low risk	High risk	Unclear	Low risk	High risk
Mendelson [51]	Unclear	Unclear	High risk	Unclear	Low risk	High risk
O’Mahen [55]	Low risk	Low risk	High risk	Low risk	Low risk	Low risk
Conoley [49]	Unclear	Unclear	High risk	Unclear	Low risk	High risk
Eggert [45]	Unclear	Unclear	High risk	Unclear	Low risk	High risk
Masia-Warner [52]	Unclear	Unclear	High risk	Low risk	Low risk	High risk
Interian [56]	Low risk	Unclear	High risk	Unclear	Low risk	High risk
Solomon [70]	Unclear	Unclear	High risk	Low risk	Low risk	High risk
Aberg-Wistedt [71]	Unclear	Unclear	High risk	Unclear	Low risk	High risk
Atkinson [67]	Unclear	Unclear	High risk	Unclear	Low risk	High risk
Terzian [73]	Unclear	Low risk	High risk	Unclear	Low risk	High risk
Hasson-Ohayon [68]	Unclear	Unclear	High risk	Unclear	Low risk	High risk
Rivera [77]	Unclear	Low risk	High risk	Low risk	Low risk	Low risk
Solomon [74]	Unclear	Unclear	High risk	Low risk	Low risk	High risk
Marzillier [75]	Low risk	Low risk	High risk	Unclear	Low risk	High risk
Stravynski [72]	Unclear	Unclear	High risk	Unclear	Low risk	High risk
Bøen [69]	Low risk	Low risk	High risk	Unclear	Low risk	High risk
Cole [76]	Low risk	Low risk	High risk	Low risk	Low risk	High risk
Schene [89]	Unclear	Unclear	High risk	Unclear	Low risk	High risk
Castelein [86]	Low Risk	Low risk	High risk	Unclear	Low risk	High risk
Gelkopf [87]	Low risk	Unclear	High risk	Unclear	Low risk	High risk
Ammerman [88]	Unclear	Low risk	High risk	Low risk	Low risk	High risk
